# Substrate-Induced Unfolding of Protein Disulfide Isomerase Displaces the Cholera Toxin A1 Subunit from Its Holotoxin

**DOI:** 10.1371/journal.ppat.1003925

**Published:** 2014-02-06

**Authors:** Michael Taylor, Helen Burress, Tuhina Banerjee, Supriyo Ray, David Curtis, Suren A. Tatulian, Ken Teter

**Affiliations:** 1 Burnett School of Biomedical Sciences, College of Medicine, University of Central Florida, Orlando, Florida, United States of America; 2 Department of Physics, University of Central Florida, Orlando, Florida, United States of America; University of California Los Angeles, United States of America

## Abstract

To generate a cytopathic effect, the catalytic A1 subunit of cholera toxin (CT) must be separated from the rest of the toxin. Protein disulfide isomerase (PDI) is thought to mediate CT disassembly by acting as a redox-driven chaperone that actively unfolds the CTA1 subunit. Here, we show that PDI itself unfolds upon contact with CTA1. The substrate-induced unfolding of PDI provides a novel molecular mechanism for holotoxin disassembly: we postulate the expanded hydrodynamic radius of unfolded PDI acts as a wedge to dislodge reduced CTA1 from its holotoxin. The oxidoreductase activity of PDI was not required for CT disassembly, but CTA1 displacement did not occur when PDI was locked in a folded conformation or when its substrate-induced unfolding was blocked due to the loss of chaperone function. Two other oxidoreductases (ERp57 and ERp72) did not unfold in the presence of CTA1 and did not displace reduced CTA1 from its holotoxin. Our data establish a new functional property of PDI that may be linked to its role as a chaperone that prevents protein aggregation.

## Author Summary

Protein disulfide isomerase (PDI) is a luminal endoplasmic reticulum (ER) protein with related but independent oxidoreductase and chaperone activities. The molecular mechanism of PDI chaperone function remains unidentified. Here, we report that PDI unfolds upon contact with the catalytic A1 subunit of cholera toxin (CT). This unfolding event dislodges CTA1 from the rest of the multimeric toxin, which is a prerequisite for the ER-to-cytosol export of CTA1 and toxin activity against the host cell. The substrate-induced unfolding of PDI is linked to its chaperone activity. Our work has established a new property of PDI that is required for CT disassembly and provides a possible structural basis for the broader role of PDI as a chaperone that prevents protein aggregation.

## Introduction

Protein disulfide isomerase (PDI) is a member of the thioredoxin superfamily with an abb'xa'c structural organization that consists of two catalytic domains (a & a′) separated by two non-catalytic domains (b & b′) and an short x linker, along with an acidic C-terminal c extension [1]–[3]. It is mainly located in the endoplasmic reticulum (ER) where it exhibits linked but independent oxidoreductase and chaperone activities. These activities allow it to facilitate the proper folding of nascent secretory proteins as well as the disposal of terminally misfolded proteins through the quality control mechanism of ER-associated degradation (ERAD). The structure and function of PDI is regulated by its redox status: it is a dynamic, flexible molecule which assumes a compact conformation in the reduced state and a more open conformation in the oxidized state [4]–[6]. PDI thus acts as a redox-dependent chaperone in its interactions with certain substrate proteins [7]–[10].

The chaperone function of PDI is defined by its ability to prevent the aggregation of misfolded proteins [11]–[14]. The importance of this activity is highlighted by the link between PDI dysfunction and neurodegeneration: a S-nitrosylated form of PDI that cannot prevent protein aggregation is found in the brains of individuals with Parkinson's or Alzheimer's disease [15]. PDI also prevents the aggregation of α-synuclein which occurs in Parkinson's disease [16], [17]. PDI can even prevent protein aggregation when added to the substrate 40 minutes after aggregation has begun [18]. This strongly suggests the chaperone function of PDI involves something other than simply binding and masking the solvent-exposed hydrophobic amino acid residues of a misfolded protein. However, the molecular mechanism of PDI chaperone function remains unknown.

PDI also plays a key role in cholera intoxication. Cholera toxin (CT) is an AB_5_ toxin that consists of a catalytic A1 subunit, an A2 linker, and a cell-binding B pentamer (Fig. S1) [19]. It moves by vesicle carriers from the cell surface to the ER where the A1 subunit dissociates from the rest of the toxin [20]. The free A1 subunit then shifts to a disordered conformation which allows it to exploit the ERAD system for export to the cytosol [21]–[24]. The translocated pool of CTA1 interacts with host factors in the cytosol to regain an active conformation, and it avoids proteasomal degradation long enough to effectively modify its Gsα target [24]–[27].

CTA1 is anchored to CTA2 by a single disulfide bond and numerous non-covalent interactions. Reduction of the A1/A2 disulfide bond can occur at the resident redox state of the ER [28], yet reduction alone is not sufficient for holotoxin disassembly [29]: PDI must displace the reduced A1 subunit from the rest of the toxin [7], [8]. This process is essential for intoxication, as PDI-deficient cells are completely resistant to CT [7].

PDI was originally thought to actively unfold the holotoxin-associated CTA1 subunit and to thereby displace CTA1 from the rest of the toxin [8]. This model was based upon the results of a protease sensitivity assay that only provided an indirect measure of protein structure. An alternative explanation for the “unfoldase” activity of PDI was suggested by our later work which demonstrated the intrinsic instability of CTA1 will allow it to spontaneously unfold upon its separation from CTA2/CTB_5_ at physiological temperature [24]. Thus, PDI could trigger toxin unfolding simply by removing CTA1 from the CT holotoxin. Our recent biophysical analysis provided experimental support for this alternative model and demonstrated that PDI does not unfold CTA1 [7]. We also found that PDI exhibits conformation-dependent interactions with CTA1: PDI recognizes the folded conformations of CTA1 present at low temperatures and in the CT holotoxin, but it does not bind to the disordered, 37°C conformation of free CTA1 [7]. Consistent with previous reports [8], we also noted only the reduced form of PDI will interact with CT and CTA1. This interaction did not appear to involve the oxidoreductase activity of PDI, as PDI did not form mixed disulfides with CTA1 and could bind to cysteine-free CTA1 deletion constructs [7], [8].

In this work we employed a biophysical and biochemical approach to define the structural basis for PDI-mediated disassembly of the CT holotoxin. Using isotope-edited Fourier transform infrared (FTIR) spectroscopy and circular dichroism (CD), we have demonstrated that PDI unfolds upon contact with CTA1. The substrate-induced unfolding of PDI provides a molecular explanation for holotoxin disassembly: the expanded hydrodynamic radius of unfolded PDI would act as a lever to dislodge reduced CTA1 from its non-covalent association with the rest of the toxin. In support of this model, we found the displacement of reduced CTA1 from CTA2/CTB_5_ does not occur when PDI is locked in a folded conformation or when PDI chaperone function is disrupted by ribostamycin treatment. Additional drug treatments with bacitracin indicated the oxidoreductase activity of PDI is not required for holotoxin disassembly. Consistent with our model, the substrate-induced unfolding of PDI is blocked by ribostamycin but not bacitracin. Two other ER-localized oxidoreductases (ERp57 and ERp72) did not unfold in the presence of CTA1 and did not displace reduced CTA1 from its holotoxin. Substrate-induced unfolding thus appears to be a unique property of PDI that is linked to its chaperone function and could explain its ability to disrupt protein aggregation.

## Results

### PDI unfolds upon contact with CTA1

**Figure 1:**
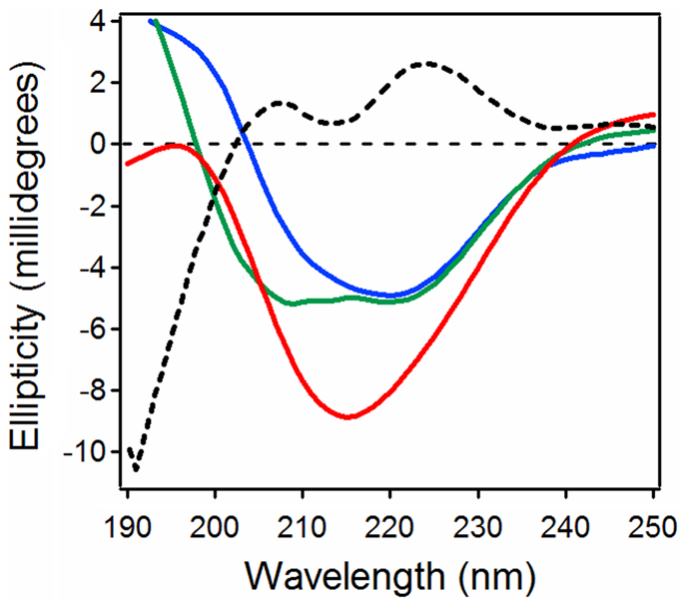
Loss of PDI structure in the presence of CTA1. Far-UV CD spectra of PDI (green), CTA1 (blue), and both proteins in the same sample at a 1∶1 molar ratio (red) are shown. The black dotted line was obtained by spectral subtraction of the individual CTA1 and PDI spectra from the spectrum of both proteins together. All measurements were taken at 10°C in pH 7.0 buffer containing 1 mM GSH.

Far-UV CD spectroscopy was used to assess conformational changes in reduced PDI and CTA1 upon their interaction at 10°C and neutral pH (Fig. 1). The spectrum of PDI alone was dominated by two components around 208 and 221 nm that can be ascribed to ππ* and nπ* backbone α-helical electronic transitions, respectively. The spectrum of CTA1 displayed a major component around 221 nm and a shoulder between 210 and 216 nm, consistent with its α/β secondary structure identified by X-ray crystallography [30], [31]. When PDI and CTA1 were combined in an equimolar ratio, the resulting spectrum was different from the sum of the two individual spectra of PDI and CTA1, suggesting that protein-protein interactions result in conformational changes in either CTA1, PDI, or both proteins. The spectral difference (i.e., [CTA1+PDI] - [CTA1] - [PDI]) revealed two well defined peaks at 207 and 223 nm, as well as a deep minimum just above 190 nm, indicating a significant loss in the α-helical structure and a gain in the unordered structure. Loss of the PDI “double minima” α-helical signature from the spectrum of PDI+CTA1 combined sample implies that significant conformational changes occur in PDI. However, the spectral overlap of CD signals generated by both proteins prevents unambiguous assignment of the structural changes to one or the other protein. To resolve individual conformational changes in PDI and CTA1 upon their interaction, we used isotope-edited FTIR spectroscopy as described below.

Isotope-edited FTIR spectroscopy allows the conformation of a protein to be monitored in the presence of a second, ^13^C-labeled protein [32]–[34]. ^13^C labeling does not alter the conformation of a protein. However, the heavier nuclear mass of the stable ^13^C isotope generates a spectral downshift which allows the FTIR spectrum of a ^13^C-labeled protein to be resolved from the spectrum of an unlabeled protein. The structures of both unlabeled and labeled proteins can thus be determined with isotope-edited FTIR spectroscopy. This technique allowed us, for the first time, to specifically and directly monitor the conformation of CTA1 in the presence of PDI. Our studies demonstrated that PDI does not unfold CTA1 [7].

**Figure 2:**
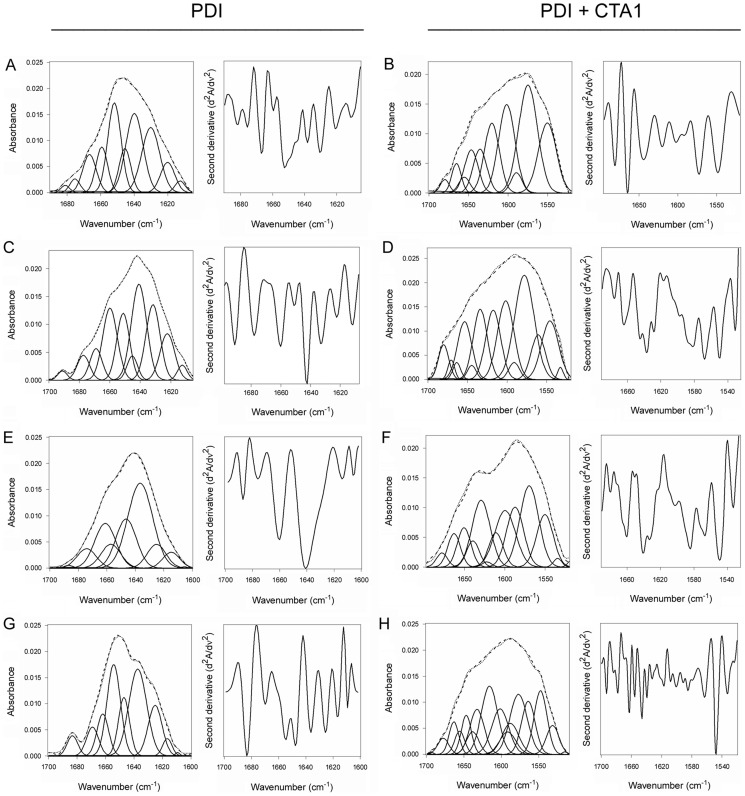
Structure of PDI in the absence or presence of CTA1. Curve fitting (left panels) and second derivatives (right panels) for the FTIR spectrum of PDI recorded in the absence (**A**, **C**, **E**, **G**) or presence (**B**, **D**, **F**, **H**) of ^13^C-labeled CTA1 are shown. For curve fitting, the dotted line represents the sum of all deconvoluted components (solid lines) from the measured spectrum (dashed line). Unless otherwise noted, all experiments were performed with sodium borate buffer (pH 7.0) containing 1 mM GSH. (**A**, **B**) PDI structure at 10°C. (**C**, **D**) PDI structure at 10°C in the absence of reductant. (**E**, **F**) PDI structure at 37°C. (**G**, **H**) PDI structure at 37°C in pH 6.5 buffer.

Here, we used isotope-edited FTIR spectroscopy to examine the impact of toxin binding on the structure of PDI (Fig. 2). Our data indicated that PDI unfolds after it contacts CTA1. In the absence of CTA1, the 10°C structure of PDI exhibited a folded conformation with 37% α-helical and 42% β-sheet content (Fig. 2A, Table 1). These percentages were consistent with the secondary structural content predicted from the crystal structure of PDI [35]. In the presence of CTA1 at 10°C, reduced PDI lost substantial α-helical and β-sheet content (Fig. 2B). The percentage of irregular PDI structure rose from 12% in the absence of CTA1 to 42% in the presence of CTA1 (Table 1). The toxin-induced loss of PDI structure did not occur in the absence of GSH (Fig. 2C–D, Table 1), which confirmed the specificity of our data: only reduced PDI can bind to CTA1 [7], [8].

**Table 1:** Toxin-induced unfolding of PDI.

	% of Protein Structure			
Condition	α-Helix	β-Sheet	Irregular	Other
PDI				
10°C	37±4	42±2	12±3	9±2
10°C+CTA1	11±1	29±3	42±4	18±2
10°C, no GSH	31±4	45±3	8±2	15±4
10°C, no GSH+CTA1	44±2	41±1	7±3	8±4
10°C EDC treatment	32±2	39±2	16±1	13±4
10°C EDC treatment+CTA1	33±4	49±1	12±3	5±5
10°C bacitracin treatment	41±2	34±3	23±1	2±4
10°C bacitracin treatment+CTA1	16±4	33±2	37±1	14±2
10°C ribostamycin treatment	36±3	41±1	18±4	6±1
10°C ribostamycin treatment+CTA1	44±1	36±2	9±2	10±3
37°C	30±3	42±1	24±4	4±1
37°C+CTA1	25±2	44±3	18±4	14±1
37°C, pH 6.5	30±1	43±3	18±2	9±2
37°C+CTA1, pH 6.5	12±4	26±3	40±4	21±1
10°C+CTA1, 37°C measurement	35±3	39±3	13±1	12±2
37°C+CT holotoxin, 1 minute	27±3	22±4	40±1	10±1
37°C+CT holotoxin, 25 minutes	30±4	38±1	17±2	16±±4
ERp57				
10°C	49±2	39±4	7±3	5±1
10°C+CTA1	46±3	36±4	11±2	8±3
ERp72				
10°C	43±2	20±2	31±2	6±1
10°C+CTA1	48±4	21±3	22±4	9±2

**Figure 3:**
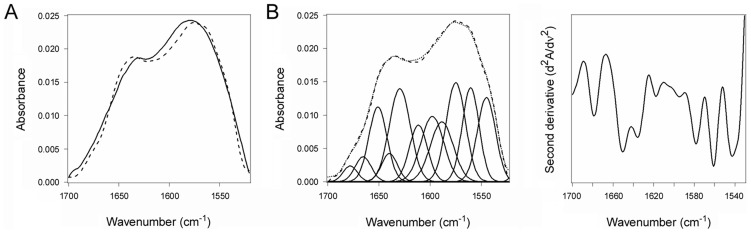
Refolding of disordered PDI. PDI was placed at 10°C in sodium borate buffer (pH 7.0) containing ^13^C-labeled CTA1 and 1 mM GSH. The temperature was then raised to 37°C for 60 min. (**A**) The FTIR spectra of PDI+CTA1 were recorded at 10°C (solid line) and at 37°C (dotted line). (**B**) Curve fitting (left panel) and second derivatives (right panel) for the FTIR spectrum of PDI at 37°C are shown. For curve fitting, the dotted line represents the sum of all deconvoluted components (solid lines) from the measured spectrum (dashed line).

**Figure 4:**
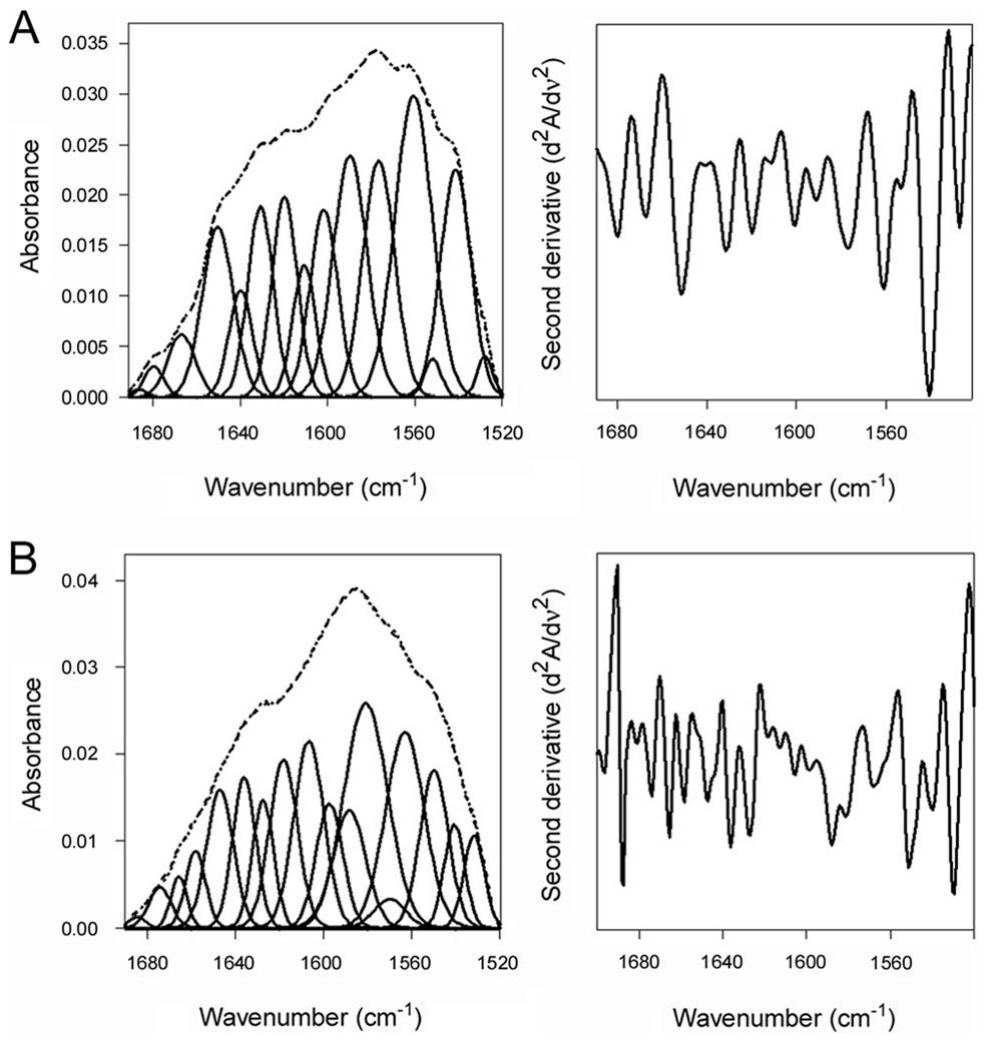
Unfolding and refolding of PDI upon interaction with the CT holotoxin. ^13^C-labeled PDI was placed at 37°C in sodium borate buffer (pH 7.0) containing 1 mM GSH and the CT holotoxin. FTIR spectra were then recorded after 1 min (**A**) or 25 min (**B**). Curve fitting (left panels) and second derivatives (right panels) for the FTIR spectrum of PDI are shown. For curve fitting, the dotted line represents the sum of all deconvoluted components (solid lines) from the measured spectrum (dashed line).

Deconvolution of the conformation-sensitive amide I bands from FTIR data presented in Fig. 2, 3, 4, 6, 8, S2, and S3 was used to calculate the percentages of PDI, ERp57, or ERp72 structure under the stated conditions. Unless otherwise indicated, all conditions included GSH in pH 7.0 buffer. The averages ± standard deviations from three or four separate curve fitting iterations are shown.

An additional set of FTIR experiments were performed at 37°C to further validate our findings. Reduced PDI does not bind to CTA1 at 37°C in pH 7.0 buffer [7] and did not exhibit an increase in irregular structure when incubated with CTA1 under this condition (Fig. 2E–F, Table 1). However, reduced PDI can bind to CTA1 at 37°C in pH 6.5 buffer [7]. Acidified medium stabilizes the CTA1 polypeptide, allowing it to retain a substantial amount of its secondary structure at physiological temperature [21]. Thus, as expected, PDI shifted to a disordered conformation with 40% irregular structure when mixed with CTA1 at 37°C in pH 6.5 buffer (Fig. 2G–H, Table 1). These collective observations demonstrated the unfolding of PDI resulted from its physical interaction with CTA1.

### PDI unfolding is reversible

The substrate-induced unfolding of PDI was a reversible event. When CTA1 was added to reduced PDI at 10°C, the folded conformation of PDI shifted to a disordered state (Fig. 2A–B, Table 1). However, PDI returned to a folded conformation upon warming the PDI/CTA1 complex to 37°C (Fig. 3, Table 1). CTA1 unfolds at 37°C, and this unfolding event displaces its PDI binding partner [7]. The displacement of reduced PDI from CTA1 at physiological temperature thus allowed PDI to regain a folded structure.

**Figure 5:**
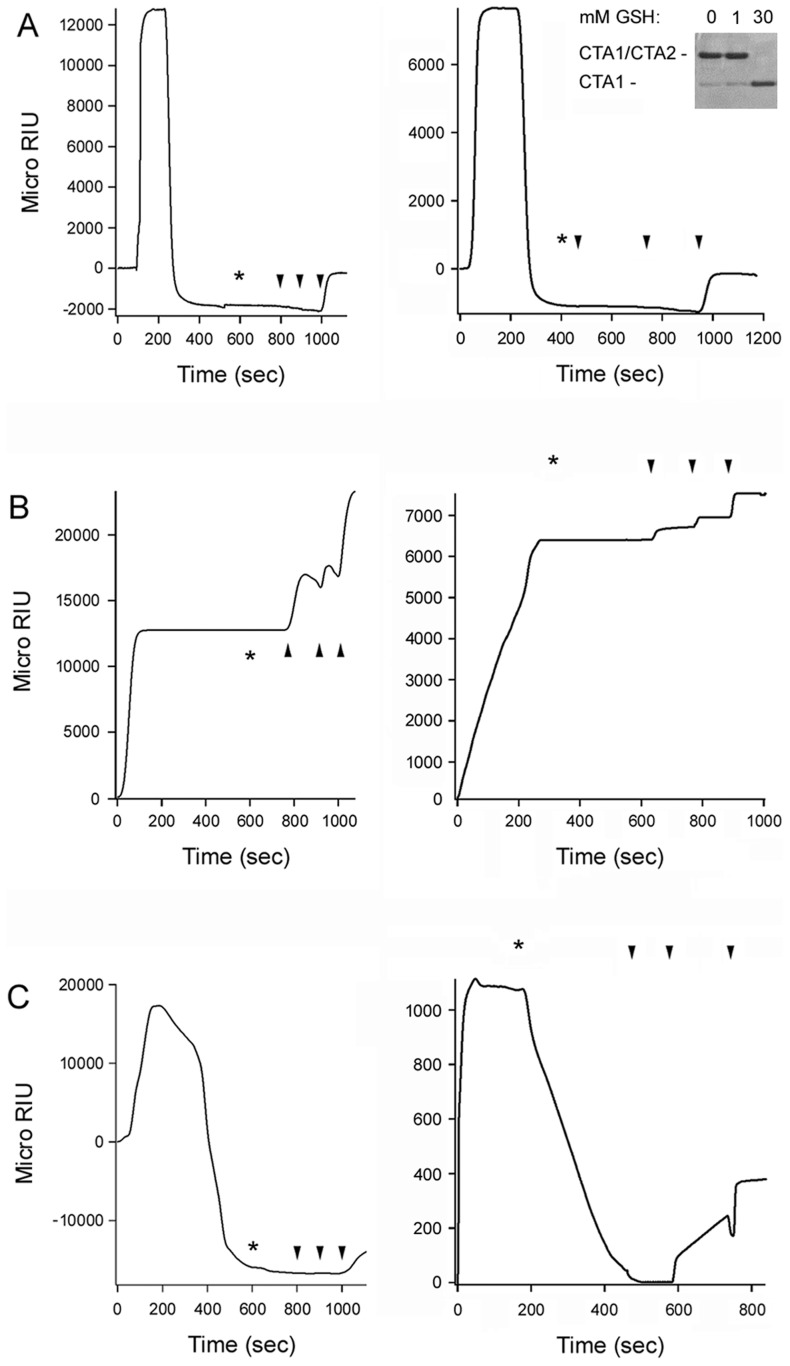
PDI unfolding but not oxidoreductase activity is required for disassembly of the CT holotoxin. SPR was used to monitor the real-time PDI-mediated disassembly of CT. A baseline measurement corresponding to the mass of the sensor-bound CT holotoxin established the 0 MicroRIU signal. The time course was then initiated with perfusion of PDI (**A**), EDC-treated PDI (**B**), or bacitracin-treated PDI (**C**) over the CT-coated sensor. The perfusion buffer contained either 30 mM GSH (left panels) or 1 mM GSH (right panels); non-reducing SDS-PAGE with Coomassie staining found the CTA1/CTA2 disulfide bond was reduced at 30 mM GSH but not 1 mM GSH (*inset*, right panel of **A**). PDI was removed from the perfusion buffer at time intervals denoted by asterisks and was replaced with sequential additions of anti-PDI, anti-CTA1, and anti-CTB antibodies as indicated by the arrowheads. One of two representative experiments is shown for each condition.

An interaction with the CT holotoxin also resulted in the unfolding and refolding of PDI (Fig. 4, Table 1). For this experiment, ^13^C-labeled PDI was mixed with the CT holotoxin at 37°C and pH 7.0. The holotoxin-associated CTA1 subunit maintains a folded conformation at 37°C and neutral pH [36], so reduced PDI can bind to holotoxin-associated CTA1 under physiological conditions. However, the spontaneous unfolding of CTA1 which occurs after holotoxin disassembly at 37°C results in the displacement of its PDI binding partner [7]. This process allowed us to monitor both the unfolding of PDI upon its interaction with the CT holotoxin and the refolding of PDI after its release from the dissociated CTA1 subunit. One minute after exposure to the CT holotoxin, reduced PDI exhibited a disordered conformation containing 40% irregular structure (Fig. 4A, Table 1). This was similar to the percentage of irregular structure in reduced PDI upon its binding to free CTA1 at 10°C and neutral pH or at 37°C and pH 6.5. After holotoxin disassembly, PDI could no longer interact with CTA1 and consequently returned to a folded conformation within 25 minutes of exposure to the CT holotoxin (Fig. 4B, Table 1). As seen from Table 1, the conformational changes in PDI between 1 and 25 minutes of its interaction with the CT holotoxin at 37°C involve an increase in the β-sheet fraction by 16% and an increase in the α-helical structure by only 3%, implying that the refolding of PDI begins with a gain of α-helical structure which is followed by a gain of β-sheet structure. The PDI-mediated displacement of reduced CTA1 from the CT holotoxin and subsequent release of PDI from the dissociated, unfolded CTA1 polypeptide are both extremely rapid events ([7], see also Fig. 5). Our data suggest these events had already occurred within 1 minute of combining PDI with CT, and the process of PDI refolding was already underway at our first time point. Technical limitations prevented the measurement of PDI structure before 1 minute of incubation with CT. Nonetheless, the data from Figures 2–4 collectively demonstrated that PDI unfolding occurs upon binding to either free CTA1 or holotoxin-associated CTA1, and the results further indicated that PDI will return to a folded state after the release of its bound substrate.

### PDI unfolding is required for holotoxin disassembly

The unfolding of PDI provides a molecular basis for the PDI-mediated disassembly of CT: unfolding will expand the hydrodynamic radius of PDI, thus acting as a wedge to dislodge reduced CTA1 from its non-covalent association with CTA2/CTB_5_. To test this model, we locked PDI in a folded conformation by treating it with 400 mM EDC. Previous work has shown this “zero-length” intramolecular cross-linker will activate carboxylic side chains for reaction with nearby primary amines on lysine residues [37], [38]. A standard cross-linking molecule is absent from this process, so the reactive side chains can only act on intramolecular targets. SDS-PAGE and size exclusion chromatography were used to confirm the absence of PDI dimers or oligomers after EDC treatment (Fig. S2A–B). Isotope-edited FTIR spectroscopy further demonstrated that EDC-treated PDI did not unfold in the presence of CTA1 (Fig. S2C–D, Table 1): the secondary structure content of EDC-treated PDI in either the absence or presence of CTA1 was similar to the secondary structure content of the isolated, untreated PDI polypeptide.

To examine the functional role of PDI unfolding in toxin disassembly, untreated PDI and EDC-treated PDI were perfused over a surface plasmon resonance (SPR) sensor coated with the CT holotoxin (Fig. 5). A functional interaction between PDI and the toxin will result in the displacement of CTA1 from the SPR sensor and a corresponding drop in the refractive index unit (RIU) below the baseline value corresponding to the mass of the initial sensor-bound holotoxin [7]. When PDI was perfused over the CT-coated sensor under reducing conditions, we detected a rapid rise in RIU which was indicative of PDI binding to the toxin. This was followed by a drop in the RIU signal to a point below the initial baseline value (Fig. 5A). Identical results were obtained with either 30 mM GSH (left panel) or 1 mM GSH (right panel) in the perfusion buffer; non-reducing SDS-PAGE with Coomassie staining demonstrated the CTA1/CTA2 disulfide bond is reduced at 30 mM GSH but not 1 mM GSH (Fig. 5A, right panel inset). The loss of signal around 200 seconds occurred even though PDI was still present in the perfusion buffer, suggesting that both PDI and CTA1 had been lost from the sensor. Sequential perfusions of anti-PDI, anti-CTA1, and anti-CTB antibodies over the PDI-treated slide confirmed this interpretation: only the anti-CTB antibody gave a positive response (Fig. 5A). Perfusion of an anti-KDEL antibody over a PDI-treated sensor also gave a positive response (not shown), which indicated the KDEL-containing CTA2 subunit remained associated with CTB_5_ after the release of CTA1. This observation was consistent with previous reports [7], [39], and it demonstrated that PDI specifically removes CTA1 from the sensor-bound CTA2/CTB_5_ complex.

EDC-treated PDI bound tightly to the CT holotoxin under reducing conditions but did not displace CTA1 from CTA2/CTB_5_ (Fig. 5B). Identical results were obtained with either 30 mM GSH (left panel) or 1 mM GSH (right panel) in the perfusion buffer. Positive signals for the anti-PDI, anti-CTA1, and anti-CTB antibodies demonstrated that EDC-treated PDI remained associated with the intact CT holotoxin after removal from the perfusion buffer. The locked conformation of PDI thus exhibited a high affinity interaction with CT, but it lacked the mechanism required to separate CTA1 from the rest of the toxin. Our FTIR data strongly suggested this missing mechanism involves the toxin-induced unfolding of PDI.

Intramolecular cross-linking could disrupt the enzymatic activity of PDI, although previous work has suggested an oxidoreductase function is not required for PDI to displace CTA1 from CTA2/CTB_5_. To confirm this observation, bacitracin-treated PDI was perfused over a CT-coated SPR sensor under reducing conditions. Bacitracin is a peptide antibiotic that inhibits the reductive activity of PDI [40], but it did not inhibit the toxin-induced unfolding of PDI as assessed by isotope-edited FTIR spectroscopy (Fig. S3A–B, Table 1). Bacitracin-treated PDI could displace reduced CTA1 from its non-covalent association with CTA2/CTB_5_ (Fig. 5C, left panel), but it could not separate CTA1 from the rest of the toxin when the CTA1/CTA2 disulfide bond was intact (Fig. 5C, right panel). In contrast, untreated PDI could mediate the disassembly of a CT holotoxin with an intact disulfide bond [7], [8] (Fig. 5A, right panel). These results indicated that the oxidoreductase activity of PDI could cleave the CTA1/CTA2 disulfide bond and that this activity was inhibited by bacitracin. Thus, bacitracin-treated PDI did not require an enzymatic function to dislodge reduced CTA1 from its non-covalent association with the rest of the toxin. By extension, the inability of EDC-treated PDI to displace reduced CTA1 from its holotoxin (Fig. 5B, left panel) could not be attributed to a loss of enzymatic function.

### The toxin-induced unfolding of PDI is related to its chaperone function

**Figure 6:**
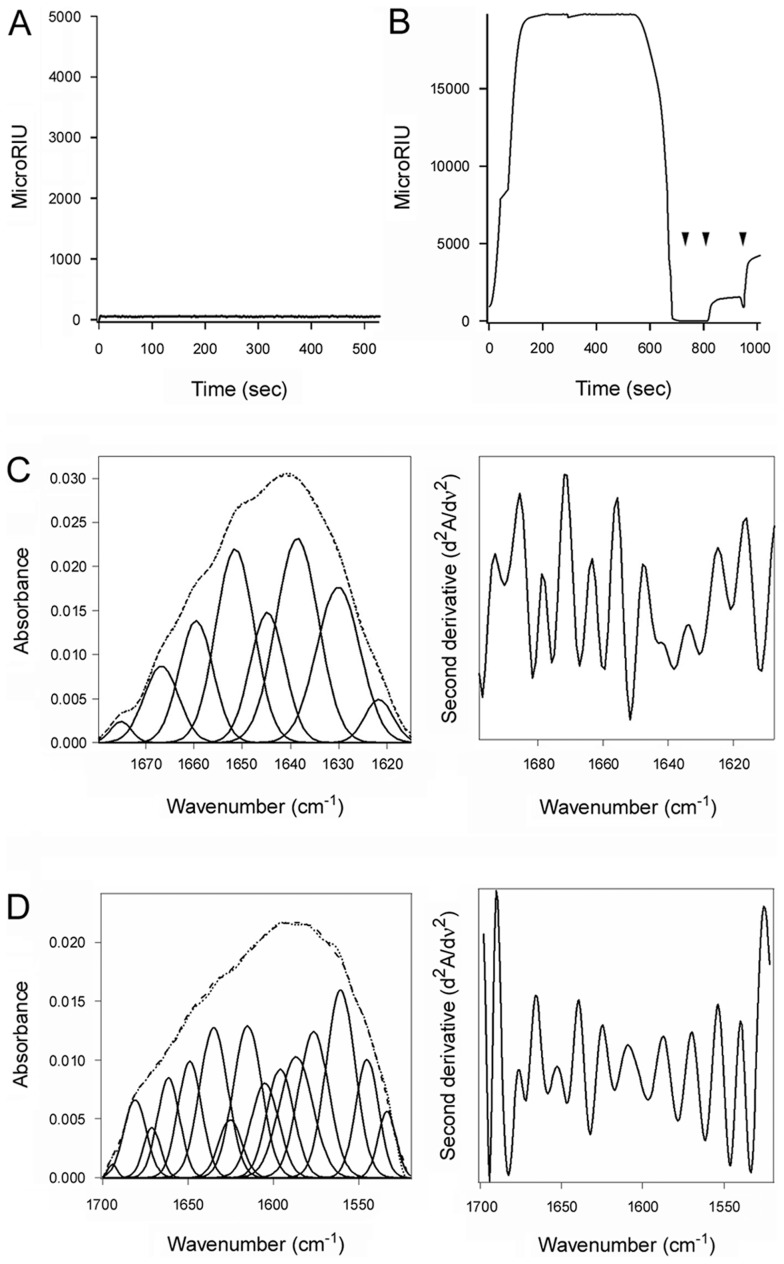
The chaperone activity of PDI is required for disassembly of the CT holotoxin. (**A**, **B**) A baseline SPR measurement corresponding to the mass of the sensor-bound CT holotoxin established the 0 MicroRIU signal. The time course was then initiated with perfusion of S-nitrosylated PDI (**A**) or ribostamycin-treated PDI (**B**) over the CT-coated sensor in buffer containing 30 mM GSH. In (**B**), PDI was removed from the perfusion buffer after 600 sec and replaced with sequential additions of anti-PDI, anti-CTA1, and anti-CTB antibodies as indicated by the arrowheads. One of two representative experiments is shown for each condition. (**C**, **D**) Curve fitting (left panels) and second derivatives (right panels) for the FTIR spectrum of ribostamycin-treated PDI recorded in the absence (**C**) or presence (**D**) of ^13^C-labeled CTA1 are shown. For curve fitting, the dotted line represents the sum of all deconvoluted components (solid lines) from the measured spectrum (dashed line).

PDI can, independently of its oxidoreductase function, act as a chaperone to prevent the aggregation of misfolded proteins [12], [13]. To determine whether the toxin-induced unfolding of PDI was related to its role as a chaperone, we used S-nitrosylation and ribostamycin to disrupt the chaperone activity of PDI [15], [41]. Nitrosylated PDI and ribostamycin-treated PDI were then used in our toxin disassembly assay. As shown in Fig. 6A, nitrosylated PDI could not bind to the CT holotoxin. The inability of nitrosylated PDI to prevent protein aggregation [15], [42] thus appears to result from the loss of substrate binding.

Ribostamycin-treated PDI could bind to the CT holotoxin but could not separate reduced CTA1 from CTA2/CTB_5_ (Fig. 6B). Removal of ribostamycin-treated PDI from the perfusion buffer resulted in a rapid drop in RIU to the initial baseline value corresponding to the mass of the CT holotoxin. Anti-PDI, anti-CTA1, and anti-CTB antibody controls confirmed that ribostamycin-treated PDI had dissociated from the intact CT holotoxin. Furthermore, isotope-edited FTIR spectroscopy demonstrated that ribostamycin-treated PDI did not unfold in the presence of CTA1 (Fig. 6C–D, Table 1). The loss of chaperone activity for ribostamycin-treated PDI thus corresponded to an inhibition of both PDI unfolding and holotoxin disassembly.

### Toxin resistance results from the loss of PDI chaperone function

**Figure 7:**
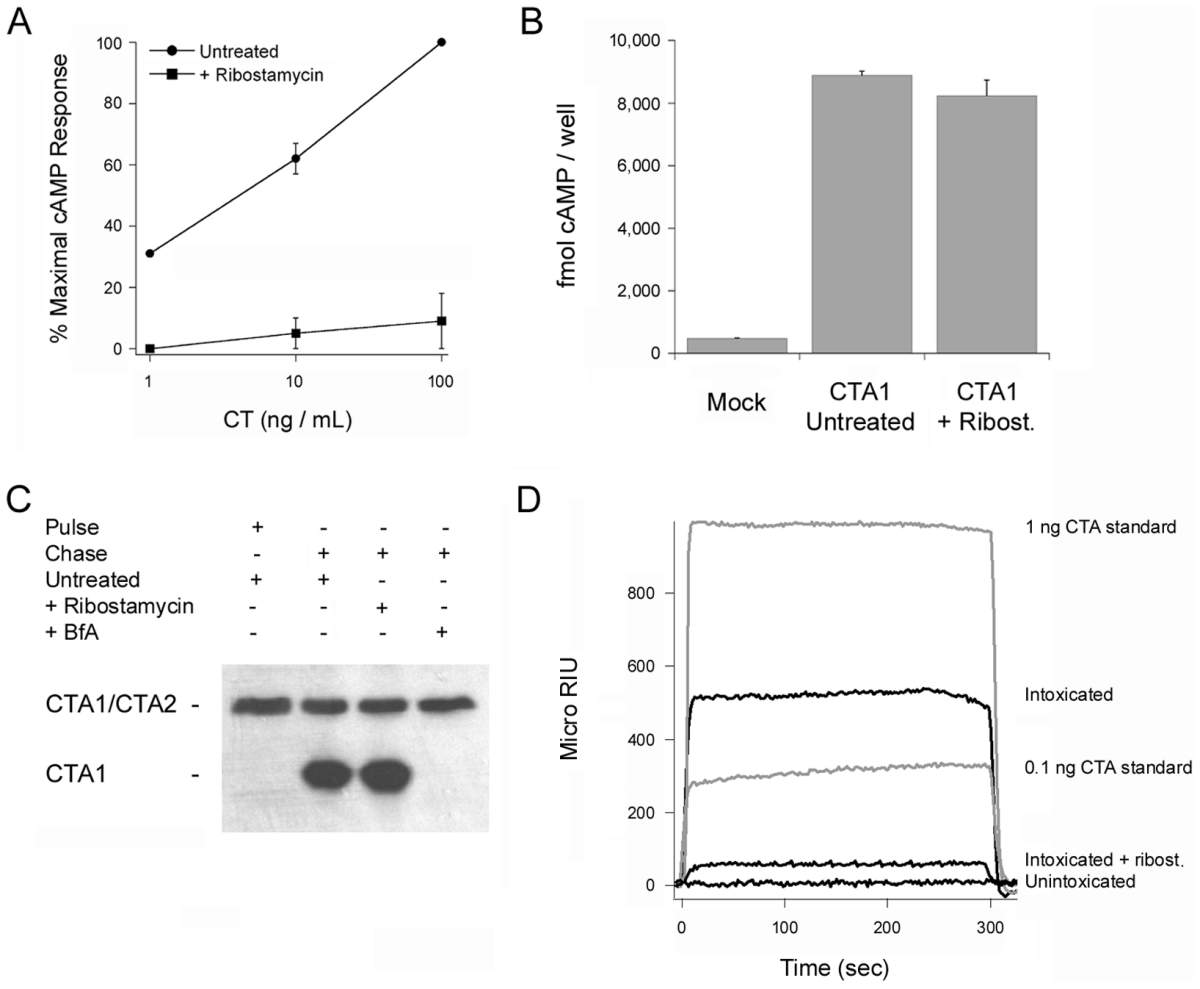
The chaperone activity of PDI is required for CT intoxication. (**A**) Untreated and ribostamycin-treated CHO cells were challenged with the stated concentrations of CT for 2 hr before cAMP levels were quantified. The averages ± ranges of 2 independent experiments with triplicate samples are shown. (**B**) CHO cells were transfected with a plasmid encoding a CTA1 construct that is co-translationally inserted into the ER. Dislocation of this CTA1 construct back to the cytosol of untreated or ribostamycin-treated cells was detected by the rise in intracellular cAMP at 4 hr post-transfection. Cells transfected with an empty plasmid (Mock) were used to establish the resting levels of cAMP. Data are presented as the averages ± standard deviations of three replicate samples per condition. One of three representative experiments is shown. (**C**) CHO cells were pulse-labeled at 4°C with 1 µg/mL of CT. Untreated or drug-treated cells were then chased in toxin-free medium for 2 hr at 37°C. Membrane fractions from digitonin-permeabilized cells were resolved by non-reducing SDS-PAGE and probed by Western blot with an anti-CTA antibody. (**D**) Untreated or ribostamycin-treated CHO cells were pulse-labeled at 4°C with 1 µg/mL of CT and then chased in toxin-free medium for 2 hr at 37°C. Cytosolic fractions from digitonin-permeabilized cells were then perfused over an SPR sensor coated with an anti-CTA1 monoclonal antibody. Known quantities of CTA were perfused over the sensor as positive controls, and the cytosolic fraction from unintoxicated cells was perfused over the sensor as a negative control. At the end of each perfusion, bound ligand was stripped from the sensor slide.

Cells treated with 50 µM ribostamycin were almost completely resistant to CT (Fig. 7A). However, no protective effect was observed in cells transfected with a plasmid encoding a CTA1 construct that is co-translationally targeted to the ER before dislocation back into the cytosol (Fig. 7B). This expression system mimics the translocation events occurring after holotoxin disassembly [43] and was used to ensure ribostamycin treatment did not affect the intoxication process downstream of PDI-mediated toxin disassembly. We also found that the CTA1/CTA2 disulfide bond could be reduced in ribostamycin-treated cells (Fig. 7C). This result indicated that ribostamycin does not inhibit toxin transport to the ER, as reduction of the CTA1/CTA2 disulfide bond takes place in the ER [28], [44]. In this experiment, brefeldin A (BfA) was used a positive control to demonstrate that CTA1/CTA2 reduction does not occur when toxin transport to the ER is blocked [45]. Ribostamycin did not block CT transport to the ER or reduction of the CTA1/CTA2 disulfide bond, yet only a minimal quantity of CTA1 could be detected in the cytosol of ribostamycin-treated cells (Fig. 7D). This was consistent with the toxin-resistant phenotype of ribostamycin-treated cells and indicated ribostamycin prevents the in vivo displacement of reduced CTA1 from CTA2/CTB_5_. Collectively, our data demonstrated that ribostamycin does not disrupt (i) toxin trafficking to the ER; (ii) reduction of the CTA1/CTA2 disulfide bond in the ER; (iii) translocation of the free CTA1 subunit from the ER to the cytosol; or (iv) CTA1 activity in the cytosol. The block of intoxication in ribostamycin-treated cells was therefore most likely due to the inhibition of PDI unfolding which facilitates holotoxin disassembly (Fig. 6). This result demonstrated the critical role of PDI unfolding in the CT intoxication process.

### Toxin-induced unfolding is a unique property of PDI

**Figure 8:**
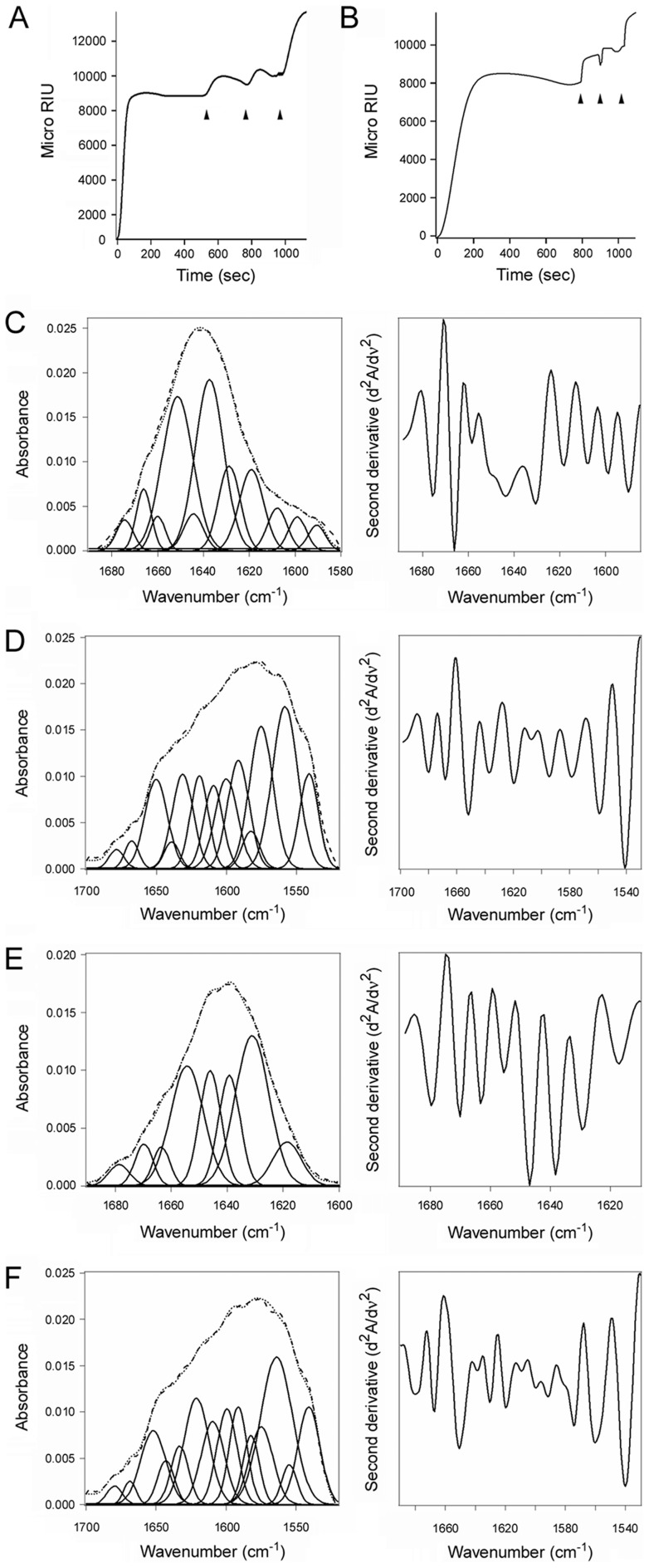
ERp57 and ERp72 do not disassemble the CT holotoxin and do not unfold upon contact with CTA1. (**A**, **B**) A baseline SPR measurement corresponding to the mass of the sensor-bound CT holotoxin established the 0 MicroRIU signal. The time course was then initiated with perfusion of ERp57 (**A**) or ERp72 (**B**) over the CT-coated sensor in buffer containing 30 mM GSH. After 300 sec (ERp57) or 350 sec (ERp72), ligand was removed from the perfusion buffer and replaced with sequential additions of anti-ERp57/anti-ERp72, anti-CTA1, and anti-CTB antibodies as indicated by the arrowheads. One of two representative experiments is shown for each condition. (**C–F**): Curve fitting (left panels) and second derivatives (right panels) for the FTIR spectrum of ERp57 (**C**, **D**) or ERp72 (**E**, **F**) recorded in the absence (**C**, **E**) or presence (**D**, **F**) of ^13^C-labeled CTA1 are shown. For curve fitting, the dotted line represents the sum of all deconvoluted components (solid lines) from the measured spectrum (dashed line).

Reduced CTA1 could not be separated from its holotoxin by ERp57 (Fig. 8A) or ERp72 (Fig. 8B), two ER-localized oxidoreductases with an overall domain structure similar to PDI [1], [2]. ERp57 and ERp72 bound to the CT holotoxin under reducing conditions, but they did not dislodge CTA1 from CTA2/CTB_5_. Indeed, as demonstrated with our antibody controls, ERp57 and ERp72 remained stably associated with the intact CT holotoxin. Additional SPR experiments documented direct binding of ERp57 and ERp72 to the CTA1 subunit (Fig. S4). ERp57 usually binds to glycosylated substrates in a complex with calnexin or calreticulin, but a direct interaction between ERp57 and its substrate has been reported as well [46]. Isotope-edited FTIR spectroscopy further demonstrated that ERp57 and ERp72 do not unfold upon contact with CTA1 (Fig. 8C–F, Table 1). The toxin-induced unfolding of PDI and the PDI-mediated displacement of CTA1 from CTA2/CTB_5_ thus appear to be unique, linked properties of PDI that are not shared by other oxidoreductases. This is consistent with the inability of CT to affect PDI-deficient cells [7]: if other resident ER oxidoreductases could perform the same function as PDI, then PDI-deficient cell lines would not be resistant to CT.

## Discussion

CT moves from the cell surface to the ER as an intact AB holotoxin. The CTA1/CTA2 disulfide bond is reduced in the ER, but this is insufficient for holotoxin disassembly: PDI must displace reduced CTA1 from the rest of the toxin. Our biophysical analysis has provided a structural explanation for this event. We have shown by isotope-edited FTIR spectroscopy and CD that PDI unfolds upon contact with CTA1. A real-time holotoxin disassembly assay demonstrated that the displacement of reduced CTA1 from CTA2/CTB_5_ does not occur when PDI is locked in a folded conformation or when the substrate-induced unfolding of PDI is blocked due to the loss of its chaperone function. However, the oxidoreductase activity of PDI was not required for this event. The toxin-induced unfolding of PDI provides a molecular basis for holotoxin disassembly: the expanded hydrodynamic radius of unfolded PDI would act as a wedge to physically displace reduced CTA1 from the rest of the toxin. ERp57 and ERp72 did not unfold in the presence of CTA1 and did not displace reduced CTA1 from its holotoxin. Substrate-induced unfolding thus appears to be a unique property of PDI.

PDI does not directly interact with CTA2 or the CTB pentamer; it only recognizes the folded conformation of the CTA1 subunit [7]. In order for PDI to dislodge CTA1 from the CT holotoxin, it must bind to a region of CTA1 near the CTA2/CTB_5_ interface. The expanded hydrodynamic radius of PDI resulting from its toxin-induced unfolding would then push against two components of the holotoxin and thereby dislodge the A1 subunit from its non-covalent association with the rest of the toxin. PDI was originally thought to interact with the C-terminal hydrophobic A1_3_ subdomain of CTA1 [47], which is distal to the CTA2/CTB_5_ interface (Fig. S1). However, binding assays with CTA1 deletion constructs have demonstrated the A1_3_ subdomain is not required for PDI-CTA1 interaction [7]. Binding instead occurred in a region of CTA1 (residues 1–133) that is, in part, proximal to CTA2/CTB_5_. The exact location of the PDI binding site on CTA1 remains to be determined, and this information is important for further elucidation of the CT disassembly mechanism. However, the current data are consistent with our model for the physical, PDI-mediated displacement of reduced CTA1 from its holotoxin.

PDI binds to the folded conformations of CTA1 that are present at low temperature and in the CT holotoxin [7]. This induces the partial unfolding of PDI (Figs. 1–2, Fig. 4, Table 1), but disordered PDI still remains associated with CTA1 [7]. The modular structure of PDI, which consists of a rigid b′ substrate binding domain flanked by other more flexible domains [2], [4], [48], likely accounts for the ability of partially disordered PDI to remain associated with its folded CTA1 partner. As shown in our recent publication, PDI is only displaced from CTA1 when the toxin unfolds [7]. CT disassembly thus appears to involve the following events: (i) the CTA1/CTA2 disulfide bond is reduced at the resident redox state of the ER [28], but CTA1 remains associated with CTA2/CTB_5_ through non-covalent interactions [29]; (ii) reduced PDI binds to holotoxin-associated CTA1 [7], [8]; (iii) the substrate-induced unfolding of PDI results in the separation of CTA1 from CTA2/CTB_5_ (this work); and (iv) the dissociated CTA1 subunit spontaneously unfolds at 37°C [24], which consequently displaces its PDI binding partner [7]. PDI regains its native conformation after release from CTA1 (Figs. 3–4). PDI and other oxidoreductases may assist reduction of the CT disulfide bond (Fig. 5A, right panel) [28], [44] as observed for other ER-translocating AB toxins [49]–[51]. However, the essential and specific role of PDI in holotoxin disassembly appears to be the physical separation of reduced CTA1 from its holotoxin.

The toxin-induced unfolding of PDI suggests a molecular mechanism for its role as a chaperone that prevents protein aggregation: by unfolding in the presence of an aggregation-prone substrate, PDI would act as a lever to dislodge individual proteins from the forming aggregate. With this model, treatments that block the chaperone activity of PDI should prevent the substrate-induced unfolding of PDI. S-nitrosylation and ribostamycin represent two such conditions, as it has already been shown that S-nitrosylated PDI and ribostamycin-treated PDI can no longer prevent protein aggregation [15], [41]. S-nitrosylation and ribostamycin treatment also blocked the PDI-mediated disassembly of CT. In the case of S-nitrosylation, the inhibition of toxin disassembly resulted from the loss of substrate binding. Ribostamycin-treated PDI could bind to CT, but it did not undergo substrate-induced unfolding. Many chaperones prevent protein aggregation by a simple physical mechanism that involves binding and masking the exposed hydrophobic amino acid residues of a disordered protein. Given that ribostamycin-treated PDI could still bind to CTA1, it is unlikely that ribostamycin disrupts the chaperone function of PDI through an inhibition of substrate binding. This strongly suggests the chaperone function of PDI involves an activity in addition to substrate binding; we propose this activity is linked to the substrate-induced unfolding of PDI. Consistent with this model, we also demonstrated that EDC-treated PDI binds to CTA1 but does not unfold in the presence of CTA1 and does not displace reduced CTA1 from CTA2/CTB_5_.

Bacitracin inhibited the enzymatic activity of PDI, but it did not affect the toxin-induced unfolding of PDI and did not prevent the displacement of reduced CTA1 from CTA2/CTB_5_. This again suggested that the substrate-induced unfolding of PDI is linked to its chaperone function, as unfolding was blocked by ribostamycin (an inhibitor of PDI chaperone function) but not bacitracin (an inhibitor of PDI oxidoreductase activity). Since holotoxin disassembly does not require the oxidoreductase function of PDI, the inhibitory effects of ribostamycin and EDC cannot be attributed to the potential disruption of PDI enzyme activity.

Our work has established a new functional property of PDI that is linked to its role as a chaperone. This property of substrate-induced unfolding would not have evolved for the benefit of a bacterial pathogen. There must therefore be a normal, physiological role for PDI unfolding. We propose the action of PDI in CT disassembly is related to its established role as a chaperone that prevents protein aggregation: in both cases, the expanded hydrodynamic radius of unfolded PDI would act as a wedge to disrupt non-covalent macromolecular complexes. This event could also be related to the structural changes that occur when PDI expands from a compact, reduced conformation to its more open, oxidized state. Thus, in addition to elucidating the molecular details of PDI-mediated toxin disassembly, our data provide a possible mechanistic basis for the known but structurally uncharacterized chaperone function of PDI.

## Materials and Methods

### Materials

Ribostamycin, PDI, BfA, GM1, GSH, CTA, S-nitrosoglutathione, and anti-CTB antibodies were purchased from Sigma-Aldrich (St. Louis, MO). Bacitracin was purchased from Calbiochem (La Jolla, CA), CT was from List Biological Laboratories (Campbell, CA), and phosphate-buffered saline (pH 7.4) with 0.05% Tween 20 (PBST) was from Medicago (Uppsala, Sweden). ERp57, the anti-ERp57 antibody, and the anti-ERp72 antibody were from Abcam (Cambridge, MA). ERp72 and the anti-PDI antibody were purchased from Enzo Life Sciences (Farmingdale, NY). The anti-CTA1 monoclonal antibody 35C2 [52] was a generous gift from Dr. Randall K. Holmes (University of Colorado School of Medicine). The pOLR130 plasmid encoding mature human PDI with an N-terminal His_6_ tag [53] was generously provided by Dr. Lloyd Ruddock (University of Oulu, Finland). Uniformly ^13^C-labeled ^13^C_6_-_D_-glucose and D_2_O were purchased from Cambridge Isotope Laboratories (Andover, MA). Uniformly ^13^C-labeled CTA1-His_6_ was produced as described in [7] and purified as described in [22].

### ^13^C labeling and purification of His_6_-PDI

*Escherichia coli* strain BL21 pLysS transformed with pOLR130 was inoculated into 5 mL M9 minimal media containing 100 µg/mL of ampicillin and was grown at 37°C with shaking. The culture was then expanded in 200 mL M9 minimal media supplemented with 100 µg/mL of ampicillin and uniformly ^13^C-labeled ^13^C_6_-D-glucose as the sole metabolic carbon source. Incubation at 37°C with shaking continued until the culture reached an O.D._600_ of 0.2–0.3. The culture was then induced with 1 mM IPTG for 4 hr at 37°C, followed by centrifugation of the cells at 3,500× g and 4°C for 20 min. The supernatant was discarded, and the cells were resuspended in extraction buffer containing 20 mM Tris-HCl (pH 7.0), 300 mM NaCl, 0.1% sodium deoxycholate, 100 µg/mL of lysozyme, and 0.1 µL/mL of DNAse. Following three freeze/thaw cycles oscillating between −80°C and 37°C, the lysed cells were centrifuged at 13,800× g and 4°C for 30 min. The supernatant was collected, syringe filtered to remove any remaining cellular debris, and supplemented with 10 µL/mL of His-PIC (Sigma-Aldrich). For every 5 mL of crude lysate, 1 mL Talon resin beads were prepared as described by the manufacturer (Clontech, Mountain View, CA). After equilibration of the resin with extraction buffer, lysate was added to the resin and agitated at room temperature for 45 min. Unbound proteins in the supernatant were removed after a 5 min room temperature spin at 700× g, and the pelleted resin was resuspended in wash buffer containing 20 mM Tris-HCl (pH 7.0), 600 mM NaCl, and 0.1% Triton X-100. Following 15 min of agitation at room temperature, the resin was centrifuged at 700× g and room temperature for 5 min. The supernatant was removed, and the resin was washed two more times with wash buffer. The washed resin was then resuspended in 20 mM Tris-HCl (pH 7.0) with 600 mM NaCl, transferred to a gravity flow column, and allowed to settle. After washing the resin bed with 5 mL 20 mM Tris-HCl (pH 7.0) containing 600 mM NaCl, His_6_-PDI was eluted from the column using 2 mL quantities of increasing imidazole concentrations (10, 15, 20, 25, 35, 40, and 50 mM). Collected fractions of 0.5 mL volume were stored at −20°C until needed. Eluted fractions as well as samples from each step of the purification process were visualized by SDS-PAGE and Coomassie staining to verify the purity of His_6_-PDI. Samples of the ^13^C-labeled protein were dialyzed in sodium borate buffer with decreasing concentrations of NaCl, lyophilized, and stored at −80°C before reconstitution in a D_2_0-based sodium borate buffer for use in FTIR spectroscopy.

### Isotope-edited FTIR spectroscopy

As previously described in [7], FTIR spectra for PDI, ERp57, or ERp72 were collected using a Jasco 4200 FTIR spectrometer at 0.964 cm^−1^ spectral resolution and a set resolution of 1 cm^−1^. Samples were prepared in a D_2_O-based 10 mM sodium borate pH* 6.6 buffer that corresponds to pH 7.0. Where indicated, a D_2_O-based 10 mM sodium borate pH* 6.1 buffer that corresponds to pH 6.5 was used for measurements at acidic pH. The buffers also contained 100 mM NaCl and, unless otherwise noted, 1 mM GSH. The 70 µL samples contained either unlabeled oxidoreductase (40 µg) or a 1∶1 molar ratio of unlabeled oxidoreductase (90 µg) and uniformly ^13^C-labeled CTA1. Studies involving PDI and the CT holotoxin used 20 µg of ^13^C-labeled His_6_-PDI and 25 µg of CT; readings for this experiment were taken from 1–25 minutes after mixing the two proteins. Absorbance spectra were determined using the matched buffer as a reference and were corrected by subtraction of water vapor contribution, smoothing, and baseline correction in the amide I region. Wavenumbers were assigned to specific protein secondary structures for unlabeled PDI as detailed in [54]: 1655±5 cm^−1^, α-helix; 1630±10 cm^−1^, β-sheet; 1644±4 cm^−1^, irregular. For ^13^C-labeled PDI, wavenumber assignments were shifted 40–50 cm^−1^. Deconvolution of protein secondary structures was performed as previously described [7]. The percentages of oxidoreductase secondary structure calculated by FTIR spectroscopy were consistent with the secondary structure content predicted from crystal structures for PDI (PDB 2B5E and PDB 4EKZ), ERp57 (PDB 3F8U), and ERp72 (reconstructed from PDBs 2DJ3, 2DJ2, 2DJ1, and 3EC3).

### Far-UV CD

Experiments were conducted with 15 µM CTA1 and equimolar PDI in 10 mM borate buffer (pH 7.0) containing 100 mM NaCl and 1 mM GSH. Measurements were recorded at 10°C using a 0.1 mm optical path-length quartz cuvette and a J-810 spectrofluoropolarimeter equipped with a PFD-425S Peltier temperature controller (Jasco Corp., Tokyo, Japan). Samples were equilibrated at 10°C for 4 min before measurement, and each spectra was averaged from 5 scans.

### SPR

To monitor disassembly of the CT holotoxin, a gold plated Reichert (Depew, NY) SPR sensor slide was coated with ganglioside GM1 and subsequently appended with CT as previously described [22]. PBST was perfused over the sensor for 10 min at 37°C with a 41 µL/min flow rate to generate a baseline RIU signal corresponding to the mass of the bound holotoxin. A PBST solution containing GSH (1 or 30 mM) and PDI, ERp57, or ERp72 (all at 100 nM final concentration) was then perfused over the sensor at 37°C and a flow rate of 41 µL/min. After removal of the oxidoreductase from the perfusion buffer, sequential additions of antibodies were perfused over the sensor at the following dilutions: anti-PDI antibody, 1∶10,000; anti-ERp57 antibody, 1∶500; anti-ERp72 antibody, 1∶500; anti-CTA1 monoclonal antibody, 1∶500; anti-CTB antibody, 1∶15,000. To detect the physical association of CTA1 with ERp57 or ERp72, each oxidoreductase was perfused at 10°C over a SPR sensor appended with CTA1-His_6_ as previously described for ARF6-CTA1 interactions [24]. All experiments were performed with a Reichert SR7000 SPR refractometer.

### Drug treatments

S-nitrosylated PDI was generated by treatment with S-Nitrosoglutathione for 20 min at room temperature. S-Nitrosoglutathione was prepared by incubating 5 mM reduced GSH with 5 mM sodium nitrate for 1 hr at room temperature. Other in vitro drug treatments involved exposing PDI for 30 min at room temperature to 0.2 mM EDC, 50 µM ribostamycin, or 0.1 mM bacitracin. A PDI stock concentration of 1.6 mg/mL was used for all treatments.

### CT intoxication assay

CHO cells grown to 80% confluency in a 24-well plate were incubated in serum-free medium with 1, 10, or 100 ng/mL of CT for 2 hr at 37°C. Toxin-challenged cells were either left untreated or were co-incubated with 50 µM ribostamycin. The cAMP content of intoxicated and unintoxicated control cells was quantified with a commercial kit (Perkin-Elmer, Boston, MA) following the manufacturer's instructions. Values obtained from unintoxicated cells were background subtracted from the results with intoxicated cells, and the experimental data were then expressed as percentages of the maximal cAMP response obtained from cells exposed to 100 ng/mL of CT in the absence of ribostamycin. Triplicate samples were used for each condition.

### CTA1 transfection/intoxication assay

CHO cells grown to 75% confluency in 6-well plates were exposed for 3 hr to a mixture of pcDNA3.1/ssCTA1 [55] and Lipofectamine according to manufacturer's instructions (Invitrogen, Carlsbad, CA). At 4 hr post-transfection, cAMP levels in untreated and ribostamycin-treated cells were quantified with a commercial kit (Perkin-Elmer). Resting levels of cAMP from cells mock transfected with the empty pcDNA3.1 vector were also recorded.

### CT transport assay

CHO cells grown to 80% confluency in 6-well plates were pulse-labeled for 30 min at 4°C in serum-free medium containing 1 µg/mL of CT. Unbound toxin was removed by washing with PBS, after which the cells were returned to toxin- and serum-free medium for a 2 hr incubation at 37°C. Chase conditions included untreated cells, cells co-incubated with 50 µM ribostamycin, and cells co-incubated with 5 µg/mL of BfA. Membrane fractions from digitonin-permeabilized cells were collected at the end of the pulse and the end of the chase conditions. Samples were resolved by non-reducing SDS-PAGE with 15% polyacrylamide gels and probed by Western blot with an anti-CTA antibody as described in [23].

### CTA1 translocation assay

As described above for the CT transport assay, untreated and ribostamycin-treated CHO cells were pulsed-labeled with CT at 4°C and chased at 37°C for 2 hr. Cytosolic fractions from digitonin-permeabilized cells were then perfused over an SPR sensor coated with the anti-CTA1 35C2 monoclonal antibody [52]. A detailed protocol for this SPR-based translocation assay has been provided in [56].

### UniProtKB/Swiss-Prot accession numbers

CT, P01555 and P01556; PDI, P17967and P07237; ERp57, P30101; ERp72, P08003.

## Supporting Information

Figure S1 **CT structure.** The catalytic 21 kDa CTA1 subunit (blue) is anchored to a CTA2 linker (red) by numerous non-covalent interactions and a single disulfide bond connecting the C-terminal A1_3_ subdomain of CTA1 (light blue) to the N-terminus of CTA2. The 5 kDa CTA2 subunit extends into the central pore of the ring-like CTB homopentamer (grey) and thus maintains extensive non-covalent contacts with CTB. A KDEL tetrapeptide is found at the C-terminus of CTA2. Separation of CTA1 from CTA2/CTB_5_ is required for the ER-to-cytosol translocation of CTA1 and optimal activation of its latent enzymatic activity. The ribbon diagram was derived from PDB 1S5F.
(TIF)

Figure S2 **Impact of EDC on the structure of PDI.** (**A**) PDI was treated with the stated concentrations of EDC for 30 min at room temperature before resolution on a non-reducing SDS-PAGE gel. One of two representative experiments is shown. (**B**) Untreated PDI (solid line) and PDI treated with 400 mM EDC (dashed line) were subjected to gel filtration with a Superdex G-75 column on an AKTA purifier. Each sample was eluted at 4°C in a buffer of 150 mM KCl and 25 mM Tris (pH 7.4) at a rate of 1 mL/min. Sample elution was detected by absorbance at 280 nm. Molecular mass standards of 150 kDa, 66 kDa, and 29 kDa eluted at 27 mL, 37 mL, and 52 mL, respectively. (**C**, **D**) PDI treated with 400 mM EDC for 30 min at room temperature was placed at 10°C in sodium borate buffer (pH 7.0) containing GSH. Curve fitting (left panels) and second derivatives (right panels) for the FTIR spectrum of EDC-treated PDI recorded in the absence (**C**) or presence (**D**) of ^13^C-labeled CTA1 are shown.
(TIF)

Figure S3 **Impact of bacitracin on the structure of PDI.** (**A**, **B**) Curve fitting (left panels) and second derivatives (right panels) for the FTIR spectrum of bactitracin-treated PDI recorded in the absence (**A**) or presence (**B**) of ^13^C-labeled CTA1 are shown. For all curve fitting, the dotted line represents the sum of all deconvoluted components (solid lines) from the measured spectrum (dashed line).
(TIF)

Figure S4 **ERp57 and ERp72 bind to the CTA1 subunit at 10°C.** ERp57 (**A**) or ERp72 (**B**) was perfused over a CTA1-coated SPR sensor slide in buffer containing 1 mM GSH. Arrowheads denote when the oxidoreductase was removed from the perfusion buffer. One of two representative experiments is shown for each condition.
(TIF)
