# Supplementary figures and images for: Substrate-Induced Unfolding of Protein Disulfide Isomerase Displaces the Cholera Toxin A1 Subunit from Its Holotoxin
Source: PLoS Pathog. 2014 Feb 6;10(2):e1003925. doi: 10.1371/journal.ppat.1003925 (PMC3916401; doi:10.1371/journal.ppat.1003925)

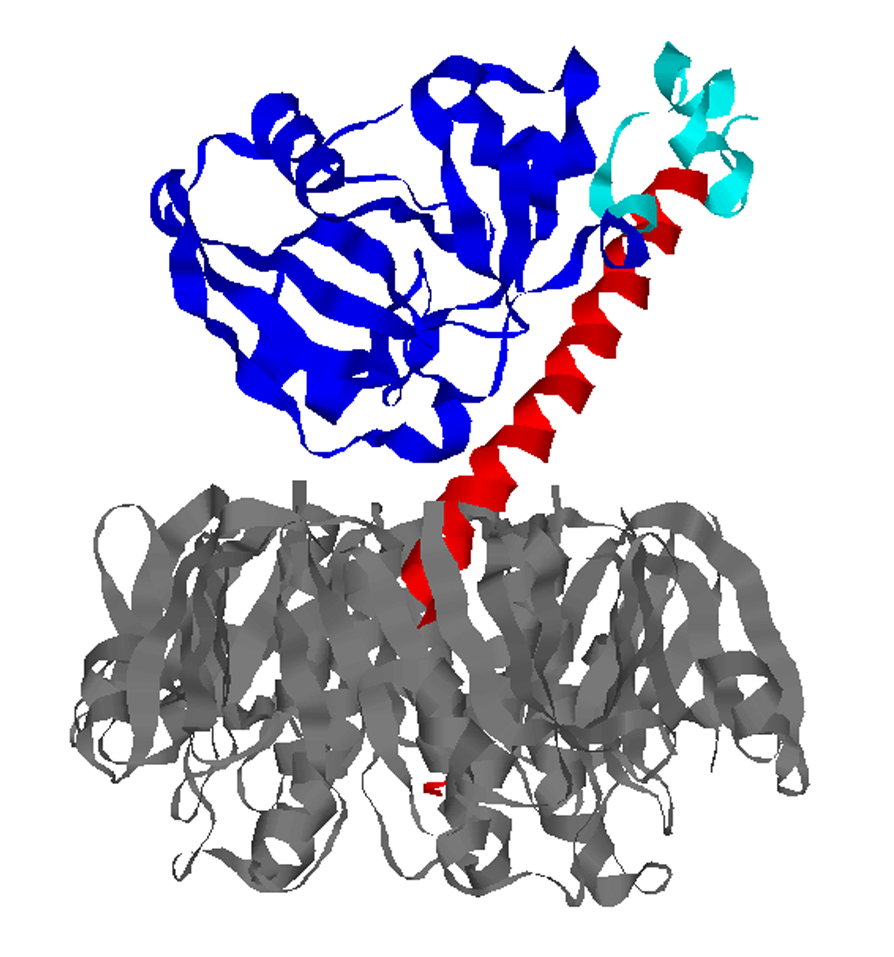

Supplement: Figure S1 — CT structure. The catalytic 21 kDa CTA1 subunit (blue) is anchored to a CTA2 linker (red) by numerous non-covalent interactions and a single disulfide bond connecting the C-terminal A13 subdomain of CTA1 (light blue) to the N-terminus of CTA2. The 5 kDa CTA2 subunit extends into the central pore of the ring-like CTB homopentamer (grey) and thus maintains extensive non-covalent contacts with CTB. A KDEL tetrapeptide is found at the C-terminus of CTA2. Separation of CTA1 from CTA2/CTB5 is required for the ER-to-cytosol translocation of CTA1 and optimal activation of its latent enzymatic activity. The ribbon diagram was derived from PDB 1S5F. (TIF) [file ppat.1003925.s001.tif]

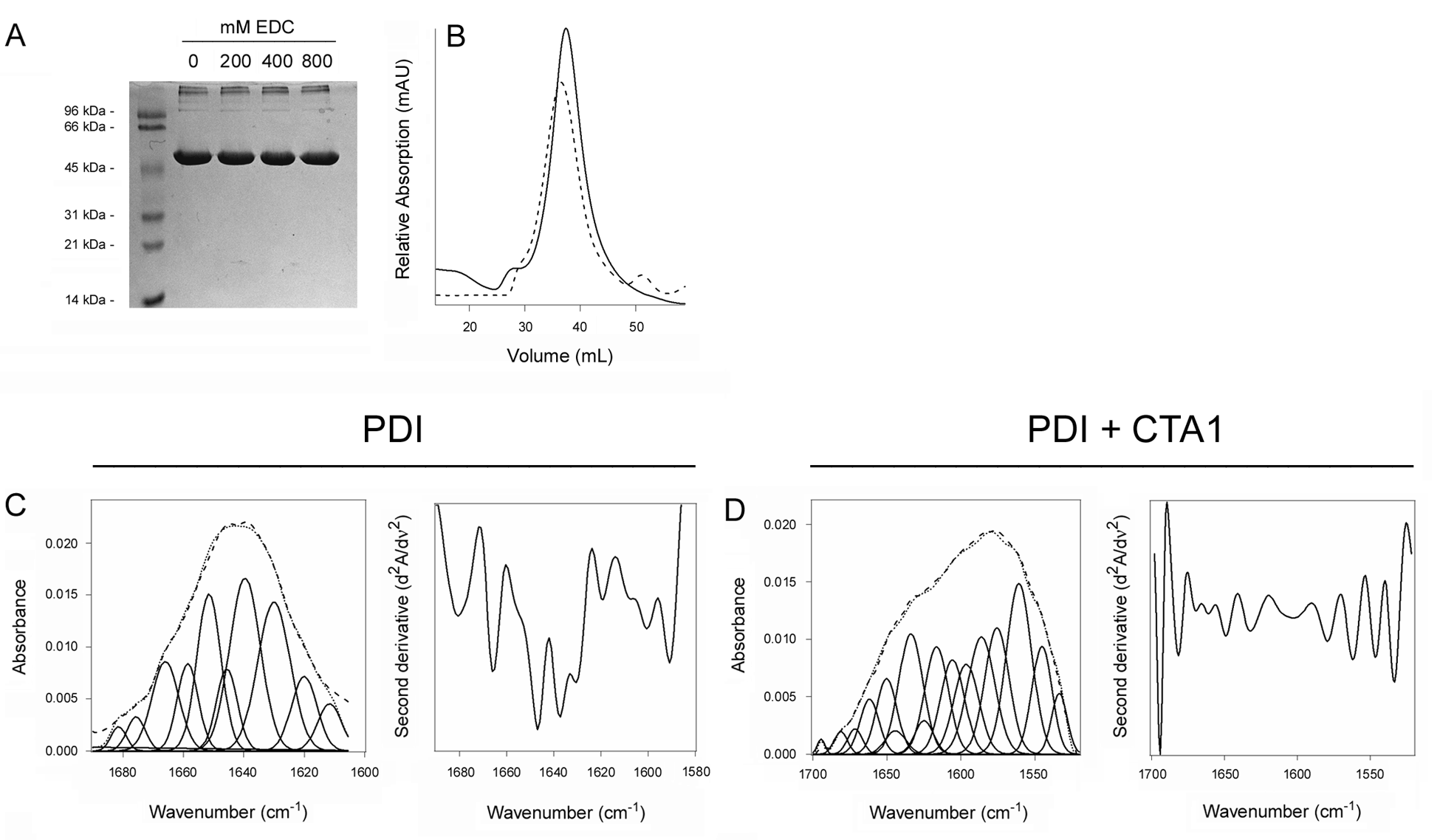

Supplement: Figure S2 — Impact of EDC on the structure of PDI. (A) PDI was treated with the stated concentrations of EDC for 30 min at room temperature before resolution on a non-reducing SDS-PAGE gel. One of two representative experiments is shown. (B) Untreated PDI (solid line) and PDI treated with 400 mM EDC (dashed line) were subjected to gel filtration with a Superdex G-75 column on an AKTA purifier. Each sample was eluted at 4°C in a buffer of 150 mM KCl and 25 mM Tris (pH 7.4) at a rate of 1 mL/min. Sample elution was detected by absorbance at 280 nm. Molecular mass standards of 150 kDa, 66 kDa, and 29 kDa eluted at 27 mL, 37 mL, and 52 mL, respectively. (C, D) PDI treated with 400 mM EDC for 30 min at room temperature was placed at 10°C in sodium borate buffer (pH 7.0) containing GSH. Curve fitting (left panels) and second derivatives (right panels) for the FTIR spectrum of EDC-treated PDI recorded in the absence (C) or presence (D) of 13C-labeled CTA1 are shown. (TIF) [file ppat.1003925.s002.tif]

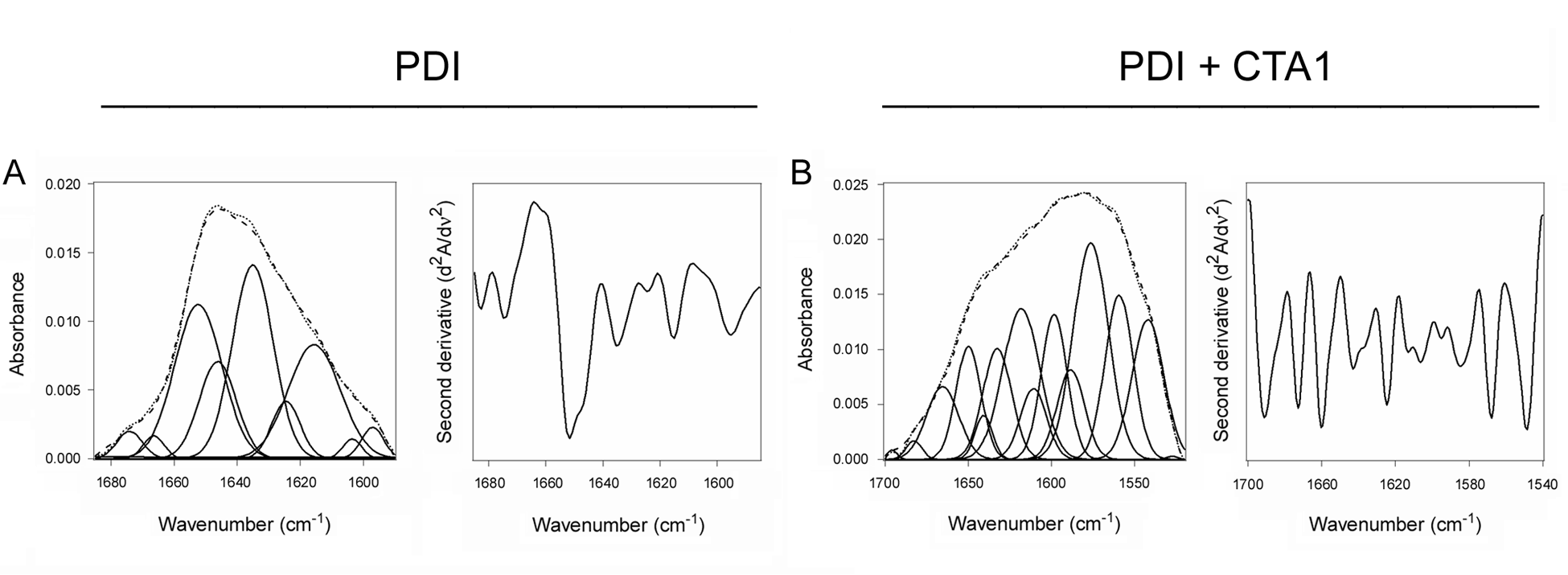

Supplement: Figure S3 — Impact of bacitracin on the structure of PDI. (A, B) Curve fitting (left panels) and second derivatives (right panels) for the FTIR spectrum of bactitracin-treated PDI recorded in the absence (A) or presence (B) of 13C-labeled CTA1 are shown. For all curve fitting, the dotted line represents the sum of all deconvoluted components (solid lines) from the measured spectrum (dashed line). (TIF) [file ppat.1003925.s003.tif]

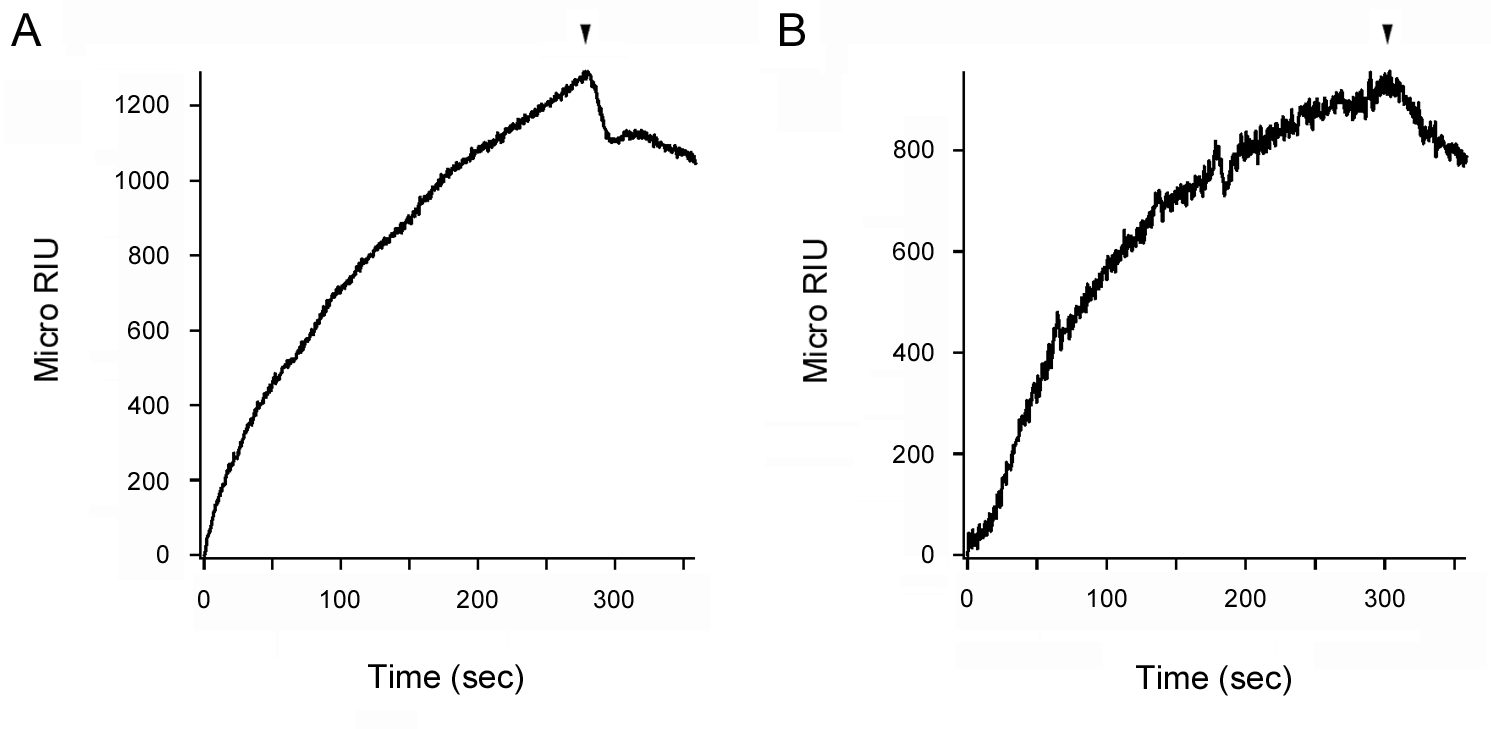

Supplement: Figure S4 — ERp57 and ERp72 bind to the CTA1 subunit at 10°C. ERp57 (A) or ERp72 (B) was perfused over a CTA1-coated SPR sensor slide in buffer containing 1 mM GSH. Arrowheads denote when the oxidoreductase was removed from the perfusion buffer. One of two representative experiments is shown for each condition. (TIF) [file ppat.1003925.s004.tif]
